# Supplementary material for: Park entrances, commonly contaminated with infective Toxocara canis eggs, present a risk of zoonotic infection and an opportunity for focused intervention
Source: PLoS Negl Trop Dis. 2025 Mar 27;19(3):e0012917. doi: 10.1371/journal.pntd.0012917 (PMC11949369; doi:10.1371/journal.pntd.0012917)
Supplement: S1 Table — (DOCX) [file pntd.0012917.s001.docx]

| **Details** | **Park 1** | **Park 2** | **Park 3** | **Park 4** | **Park 5** | **Park 6** | **Park 7** | **Park 8** | **Park 9** | **Park 10** | **Park 11** | **Park 12** |
| --- | --- | --- | --- | --- | --- | --- | --- | --- | --- | --- | --- | --- |
| Number of samples taken | 53 | 62 | 59 | 61 | 61 | 88 | 70 | 70 | 70 | 70 | 71 | 68 |
| Number of positive samples | 2 | 2 | 14 | 0 | 1 | 10 | 0 | 2 | 4 | 2 | 0 | 2 |
| % Positive samples | 3.77 | 3.22 | 23.73 | 0 | 1.64 | 11.36 | 0 | 2.86 | 5.71 | 2.86 | 0 | 2.94 |
| Number of eggs detected | 3 | 6 | 39 | 0 | 1 | 31 | 0 | 5 | 4 | 2 | 0 | 3 |
| Number of infective eggs detected | 3 | 6 | 36 | 0 | 1 | 30 | 0 | 5 | 3 | 1 | 0 | 2 |
| % Infective eggs | 100 | 100 | 92.3 | n/a | 100 | 96.8 | n/a | 100 | 75 | 50 | n/a | 66.6 |
| Mean number of eggs per sample (± SE) | 0.057 (0.042) | 0.097  (0.082) | 0.382  (0.129) | 0 | 0.016  (0.016) | 0.352  (0.181) | 0 | 0.071  (0.052) | 0.057  (0.028) | 0.029  (0.020) | 0 | 0.052  (0.038) |
| Mean number of eggs per gram of soil  (± SE) | 0.001  (0.001) | 0.002  (0.002) | 0.008  (0.003) | 0 | 0.0003  (0.0003) | 0.007  (0.004) | 0 | 0.001  (0.001) | 0.001  (0.0006) | 0.001  (0.0004) | 0 | 0.001  (0.0008) |
| Mean eggs per positive samples  (± SE) | 1.5  (0.50) | 3  (2.00) | 2.786  (0.656) | n/a | 1  (n/a) | 3.1  (1.36) | n/a | 2.5  (0.50) | 1.0  (0.00) | 1.0  (0.00) | n/a | 1.5  (0.50) |
| Mean EPG per positive samples  (± SE) | 0.30  (0.01) | 0.06  (0.04) | 0.056  (0.013) | n/a | 0.020 (n/a) | 0.062 (0.027) | n/a | 0.050  (0.010) | 0.020  (0.00) | 0.020  (0.00) | n/a | 0.030  (0.010) |

**S1 Table. Summary of results for each park**
