# Supplementary material for: Park entrances, commonly contaminated with infective Toxocara canis eggs, present a risk of zoonotic infection and an opportunity for focused intervention
Source: PLoS Negl Trop Dis. 2025 Mar 27;19(3):e0012917. doi: 10.1371/journal.pntd.0012917 (PMC11949369; doi:10.1371/journal.pntd.0012917)
Supplement: S1 File — Photographs and measurements of the eggs recovered as part of the study. (ZIP) [file pntd.0012917.s003.zip › Supplementary Information_Egg Pictures.docx]

| # | Egg ID | Photos (not to scale) | Dimensions (µm) | Ascarid primers (XZ1/NC2) | T canis primers (YY1/NC2) | Sangar sequencing result |
| --- | --- | --- | --- | --- | --- | --- |
| 1 | BG3_T1 Entrance |  | 86 x 71 | Positive  Tcanis | Positive | T. canis |
| 2 | BG7_Toxo1  BG7B_1/2 |  | 90 x 70 | Lost egg prior to PCR | Lost egg prior to PCR |  |
| 3 | BG7_Toxo2  BG7B_2/2 |  | 87 x 71 | Failed PCR | Failed PCR |  |
| 4 | R32 |  | 79 x 61 | Lost prior to PCR |  |  |
|  | R40 1/2 |  | 99 x 72 | Lost prior to PCR |  |  |
| 6 | R40 2/2 |  | 84 x 83 | Lost prior to PCR |  |  |
| 7 | FV1 1/1 |  | 88 x 87 | Failed PCR | Failed PCR |  |
| 8 | FVPG5_1/5 |  | 81 x 71 | Negative PCR | Negative PCR |  |
| 9 | FVPG5_2/5 |  | 89 x 79 | Positive | Positive | T. canis |
| 10 | FVPG5_3/5 |  | 85 x 70 | Positive | Positive | T. canis |
| 11 | FVPG5_4/5 |  | 96 x 85 | Positive | Positive | T. canis |
| 12 | FVPG5_5/5 |  | 91 x 81 | Lost prior to PCR | Lost prior to PCR |  |
| 13 | RGPG8_1/1 |  | 89 x 67 | Lost prior to PCR | Lost prior to PCR |  |
| 14 | RGE17b 1/2 | Photo lost |  |  |  |  |
| 15 | RGE17b 2/2 | Photo lost |  |  |  |  |
| 16 | RGENT1_Toxo1 |  | 91 x 77 | Positive | Positive | T. canis |
| 17 | RGENT1_Toxo2 |  | 86 x 72 | Negative PCR | Negative PCR |  |
| 18 | RGENT1_Toxo3 |  | 92 x 76 | Positive | Positive |  |
| 19 | RGENT1_Toxo4 |  | 92 x 70 | Positive | Positive |  |
| 20 | RGENT1_Toxo5 |  | 93 x 78 | PCR product on Run 3. | Negative PCR |  |
| 21 | RGENT1_Toxo6 |  | 88 x 69 | Positive | Positive |  |
| 22 | RGENT3_Toxo1 |  | 82 x 70 |  |  |  |
| 23 | RGENT4_Toxo1 |  | 85 x 73 |  |  |  |
| 24 | RGENT6_Toxo1 |  | 86 x 83 |  |  |  |
| 25 | RG_ENT_7_ 1/3 |  | 100 x 68 |  |  |  |
| 26 | RG_ENT_7_Toxo 2/3 |  | 87 x 84 |  |  |  |
| 27 | RG_ENT_7_Toxo 3/3 | Photo lost |  |  |  |  |
| 28 | RGENT_10_1/3 |  | 89 x 87 |  |  |  |
| 29 | RGENT_10_2/3 |  | 89 x 83 |  |  |  |
| 30 | RGENT_10_3/3 |  | 87 x 71 |  |  |  |
| 31 | RGEntB2 |  | 88 x 81 | Negative PCR | Negative PCR |  |
| 32 | RGEB4  _1/5 |  | 82 x 80 |  |  |  |
| 33 | RGEB4_2/5 |  | 85 x 75 |  |  |  |
| 34 | RGEB4_3/5 |  | 81 x 79 |  |  |  |
| 35 | RGEB4_4/5 |  | 89 x 76 |  |  |  |
| 36 | RGEB4_5/5 |  | 80 x 73 |  |  |  |
| 37 | RGENTB9_1/1 |  | 82 x 79 |  |  |  |
| 38 | TV36 1/1 |  | 83 x 67 |  |  |  |
| 39 | GT33 |   Incubated from January to May. | 98 x 72 |  |  |  |
| 40 | GTEA8_1/  15 |  | 89 x 83 |  |  |  |
| 41 | GTEA8_2/15 |  | 95 x 72 |  |  |  |
| 42 | GTEA8_3/15 |  | 89 x 86 |  |  |  |
| 43 | GTEA8_4/15 |  | 95 x 81 |  |  |  |
| 44 | GTEA8_5/15 |  | 94 x 74 |  |  |  |
| 45 | GTEA8_6/15 |  | 100 x 95 |  |  |  |
| 46 | GTEA8_7/15 |  | 83 x 69 |  |  |  |
| 47 | GTEA8_8/15 |  | 90 x 76 |  |  |  |
| 48 | GTEA8_9/15 |  | 92 x 73 |  |  |  |
| 49 | GTEA8_10/15 |  | 89 x 80 |  |  |  |
| 50 | GTEA8_11/15 |  | 83 x 78 |  |  |  |
| 51 | GTEA8_12/15 |  | 93 x 79 |  |  |  |
| 52 | GTEA8_13/15 |  | 85 x 76 |  |  |  |
| 53 | GTEA8_14/15 |  | 79 x 67 |  |  |  |
| 54 | GTEA8_15/15 |  | 85 x 84 |  |  |  |
| 55 | GTEA9_T1 |  |  |  |  |  |
| 56 | GTPG9_1/4 |  | 76 x 69 |  |  |  |
| 57 | GTPG9_2/4 |  | 88 x 77 |  |  |  |
| 58 | GTPG9_3/4 |  | 82 x 79 |  |  |  |
| 59 | GTPG9_4/4 |  | 87 x 66 |  |  |  |
| 60 | MR1_1/1 |  | 89 x 70 |  |  |  |
| 61 | MR6_1/9 |  | 85 x 65 |  |  |  |
| 62 | MR6_2/9 |  | 90 x 78 |  |  |  |
| 63 | MR6_3/9 |  | 94 x 81 |  |  |  |
| 64 | MR6_4/9 |  | 94 x 83 |  |  |  |
| 65 | MR6_5/9 |  | 86 x 73 |  |  |  |
| 66 | MR6_6/9 |  | 87 x 78 |  |  |  |
| 67 | MR6_7/9 |  | 84 x 83 |  |  |  |
| 68 | MR6_8/9 |  | 90 x 79 |  |  |  |
| 69 | MR6_9/9 |   Before (3/2/22) and after incubation (1/3/2) | 90 x 87 |  |  |  |
| 70 | MRG6_1/4 |  | 82 x 68 |  |  |  |
| 71 | MRG6_2/4 |  | 87 x 72 |  |  |  |
| 72 | MRG6_3/4 |  | 87 x 81 |  |  |  |
| 73 | MRG6_4/4 |  | 88 x 84 |  |  |  |
| 74 | MGTG1_1/1 | This was unembryonated but developed a motile larva following incubation | 84 x 71 |  |  |  |
| 75 | MGTG2_1/2 |  | 84 x 71 |  |  |  |
| 76 | MGTG2_2/2 |  | 90 x 84 |  |  |  |
| 77 | MGTG5_1/3 | Incubated from February to March |  |  |  |  |
| 78 | MGTG5_2/3 |   Incubated from February to March | 73 x 62 |  |  |  |
| 79 | MGTG5_3/3 |  | 93 x 81 |  |  |  |
| 80 | MGTG12_1/2 | Picture lost |  |  |  |  |
| 81 | MGTG12_2/2 | Picture lost |  |  |  |  |
| 82 | MGTR1_1/1 |  | 84 x 78 |  |  |  |
| 83 | MGTR8_1/1 |  | 88 x 84 |  |  |  |
| 84 | JT58_1/2 |  | 67 x 56 |  |  |  |
| 85 | JT58_2/2 |  | 84 x 62 |  |  |  |
| 86 | EC0_1/2 |   Incubated March - November: no development | 88 x 82 |  |  |  |
| 87 | EC30_1/1 |  | 83 x 72 |  |  |  |
| 88 | ECPG5_1/1 |  | 84 x 71 |  |  |  |
| 89 | ECPG8_1/1 |  | 92 x 81 |  |  |  |
| 90 | SA35_1/1 |   Incubated more than 1 month | 93 x 74 |  |  |  |
| 91 | SA44_1/1 |  | 90 x 86 |  |  |  |
| 92 | JT30_1/3 |  | 94 x 68 |  |  |  |
| 93 | JT30_2/3 |  | 88 x 68 |  |  |  |
| 94 | JT30_3/3 |  | 88 x 80 |  |  |  |
