# Supplementary material for: Park entrances, commonly contaminated with infective Toxocara canis eggs, present a risk of zoonotic infection and an opportunity for focused intervention
Source: PLoS Negl Trop Dis. 2025 Mar 27;19(3):e0012917. doi: 10.1371/journal.pntd.0012917 (PMC11949369; doi:10.1371/journal.pntd.0012917)
Supplement: Attachment — Submitted filename: Response to reviewers_PLOSNTD_JK.docx [file pntd.0012917.s004.docx]

*Please include the following items when submitting your revised manuscript:*

** A rebuttal letter that responds to each point raised by the editor and reviewer(s). You should upload this letter as a separate file labeled 'Response to Reviewers'. This file does not need to include responses to any formatting updates and technical items listed in the 'Journal Requirements' section below.*

** A marked-up copy of your manuscript that highlights changes made to the original version. You should upload this as a separate file labeled 'Revised Manuscript with Track Changes'.*

** An unmarked version of your revised paper without tracked changes. You should upload this as a separate file labeled 'Manuscript'.*

*If you would like to make changes to your financial disclosure, competing interests statement, or data availability statement, please make these updates within the submission form at the time of resubmission. Guidelines for resubmitting your figure files are available below the reviewer comments at the end of this letter.*

Author response – Files named and included as requested.

**Additional Editor Comments :**

*How were the twelve parks selected? How many parks were possible to be sampled from?*

The following text has been added:
“Of the 66 parks under the management of Dublin City Council, 12 parks were chosen in order to provide a geographical spread of park locations, both north and south of the river Liffey, in addition to including parks of similar size and facilities. Very small parks with few facilities were usually not sampled, unless there was no other suitable park in the area. For example, parks closer to the city centre had fewer playing field facilities, so smaller parks were included in these locations”

*In the statistical methods, please be clearer about what is meant by the ‘level of contamination’. What exactly is the response variable here? Ensure that the wording is consistent throughout from methods, results, and tables/figures.*

“Level of contamination“ changed to “numbers of Toxocara eggs” where appropriate.

*How were Kruskal-Wallis tests adjusted for ties and why?*

The following text has been added:

“The Kruskal-Wallis test was performed using Minitab, which automatically adjusts the test statistic for ties using a correction factor to maintain the validity of the chi-square approximation.”

*In the conclusion paragraph, be clear in the first sentence that you are referring to the general widespread contamination and not that this is a result from this study.*

The following text has been added to the conclusion

“In Ireland and around the world, the widespread contamination of public places with the eggs of Toxocara spp. represents a potential risk to public health”

***Journal Requirements:***

*1) Please ensure that the CRediT author contributions listed for every co-author are completed accurately and in full.*

*At this stage, the following Authors/Authors require contributions: Jason Keegan, Paul Airs, Claire Brown, Anya Rishi Dingley, Conor Courtney, Eric R Morgan, and Celia Holland. Please ensure that the full contributions of each author are acknowledged in the "Add/Edit/Remove Authors" section of our submission form.
The list of CRediT author contributions may be found here:* [*https://journals.plos.org/plosntds/s/authorship#loc-author-contributions*](https://journals.plos.org/plosntds/s/authorship#loc-author-contributions)

Added

2) Please provide an Author Summary. This should appear in your manuscript between the Abstract (if applicable) and the Introduction, and should be 150-200 words long. The aim should be to make your findings accessible to a wide audience that includes both scientists and non-scientists. Sample summaries can be found on our website under Submission Guidelines:
<https://journals.plos.org/plosntds/s/submission-guidelines#loc-parts-of-a-submission>

Author Summary:

The common roundworms of dogs and cats are also capable of infecting other animals, including humans. Infected dogs and cats can release parasite eggs into the environment, where they can develop to infectivity if the conditions are sufficiently warm and moist. Accidental ingestion of these eggs can cause difficult to diagnose illness in humans. The aim of this study was to measure the number of *Toxocara* spp. eggs in soil samples of public parks in Dublin City. We aimed to do this in a clear and repeatable manner, using freely available software to help standardise the selection of soil sampling locations, as well as providing a detailed methodology regarding how soil samples were actually taken, information which is often lacking from similar studies. In addition, we aimed to establish the species of each egg detected using PCR, to identify whether dogs or cats were likely responsible for the presence of eggs in the environment. Based on the results of this study, park entrances were the most commonly contaminated location within each park, and dogs were responsible for the majority of this contamination.

*3) We do not publish any copyright or trademark symbols that usually accompany proprietary names, eg ©,  ®, or TM  (e.g. next to drug or reagent names). Therefore please remove all instances of trademark/copyright symbols throughout the text, including:
- ® on page: 10.*

Removed

*4) Please upload all main figures as separate Figure files in .tif or .eps format. For more information about how to convert and format your figure files please see our guidelines:* 
[*https://journals.plos.org/plosntds/s/figures*](https://journals.plos.org/plosntds/s/figures)

Added

*5) We have noticed that there is a reference to supplementary table 5 on pages 20 and 21. However, there is no corresponding file uploaded to the submission. Please upload it as a separate file with the item type 'Supporting Information', or if it is no longer to be included, please remove any references to it within the manuscript.*

Corrected

*6) We have noticed that you have uploaded Supporting Information files, but you have not included a list of legends. Please add a full list of legends for your Supporting Information files after the references list.*

Added

*7) We note that your Data Availability Statement is currently as follows: "All relevant data are within the manuscript and its Supporting Information files.". Please confirm at this time whether or not your submission contains all raw data required to replicate the results of your study. Authors must share the “minimal data set” for their submission. PLOS defines the minimal data set to consist of the data required to replicate all study findings reported in the article, as well as related metadata and methods (*[*https://journals.plos.org/plosone/s/data-availability#loc-minimal-data-set-definition*](https://journals.plos.org/plosone/s/data-availability#loc-minimal-data-set-definition)*).
For example, authors should submit the following data: 
1) The values behind the means, standard deviations and other measures reported;
2) The values used to build graphs;
3) The points extracted from images for analysis..
Authors do not need to submit their entire data set if only a portion of the data was used in the reported study.
If your submission does not contain these data, please either upload them as Supporting Information files or deposit them to a stable, public repository and provide us with the relevant URLs, DOIs, or accession numbers. For a list of recommended repositories, please see* [*https://journals.plos.org/plosone/s/recommended-repositories*](https://journals.plos.org/plosone/s/recommended-repositories)*. 
If there are ethical or legal restrictions on sharing a de-identified data set, please explain them in detail (e.g., data contain potentially sensitive information, data are owned by a third-party organization, etc.) and who has imposed them (e.g., an ethics committee). Please also provide contact information for a data access committee, ethics committee, or other institutional body to which data requests may be sent. If data are owned by a third party, please indicate how others may request data access.*

Added to supporting information files.

8) Please amend your detailed Financial Disclosure statement. This is published with the article. It must therefore be completed in full sentences and contain the exact wording you wish to be published.

1) State the initials, alongside each funding source, of each author to receive each grant. For example: "This work was supported by the National Institutes of Health (####### to AM; ###### to CJ) and the National Science Foundation (###### to AM)."

2) State what role the funders took in the study. If the funders had no role in your study, please state: "The funders had no role in study design, data collection and analysis, decision to publish, or preparation of the manuscript."

Please also include the grant number in the Financial Disclosure statement.

Completed.

**Reviewers' comments:**

*Reviewer's Responses to Questions*

***Key Review Criteria Required for Acceptance?*** *As you describe the new analyses required for acceptance, please consider the following:*

***Methods*** *-Are the objectives of the study clearly articulated with a clear testable hypothesis stated?

-Is the study design appropriate to address the stated objectives?

-Is the population clearly described and appropriate for the hypothesis being tested?

-Is the sample size sufficient to ensure adequate power to address the hypothesis being tested?

-Were correct statistical analysis used to support conclusions?

-Are there concerns about ethical or regulatory requirements being met?*

*Reviewer #1: YES*

*Reviewer #2: no concerns, excellent author included actual egg sizes*

***Results*** *-Does the analysis presented match the analysis plan?

-Are the results clearly and completely presented?

-Are the figures (Tables, Images) of sufficient quality for clarity?*

*Reviewer #1: YES*

*Reviewer #2: Table 2 questions: in final 3 columns data was presented as %’s but all were entered as >100% I was confused if this was intentional*

In the table title, we mention “**Percent recovery is based on the fraction expected in 50/200g of soil, i.e. 25% of eggs added.”.** The results show we recovered more eggs from the first 50g of sieved soil than we might have expected. They may have passed through in proportion to the weight (i.e. how we estimated) or some eggs may have remained stuck to larger debris which would have resulted in a lower recovery rate. We have added further clarification to the table columns

***Conclusions*** *-Are the conclusions supported by the data presented?

-Are the limitations of analysis clearly described?

-Do the authors discuss how these data can be helpful to advance our understanding of the topic under study?

-Is public health relevance addressed?*

*Reviewer #1: YES*

*Reviewer #2: -Are the conclusions supported by the data presented? yes
-Are the limitations of analysis clearly described? yes
-Do the authors discuss how these data can be helpful to advance our understanding of the topic under study? yes
-Is public health relevance addressed? yes!*

***Editorial and Data Presentation Modifications?*** *Use this section for editorial suggestions as well as relatively minor modifications of existing data that would enhance clarity. If the only modifications needed are minor and/or editorial, you may wish to recommend “Minor Revision” or “Accept”.*

*Reviewer #1: ACCEPT*

*Reviewer #2: See Results*

***Summary and General Comments*** *Use this section to provide overall comments, discuss strengths/weaknesses of the study, novelty, significance, general execution and scholarship. You may also include additional comments for the author, including concerns about dual publication, research ethics, or publication ethics. If requesting major revision, please articulate the new experiments that are needed.*

*Reviewer #1: This manuscript provides a useful guide for others conducting similar research on the contamination of parks and public places with dog and cat feces as a means of identifying potential areas for control. the manuscript is well written, referenced and remarkably free of errors. These types of studies have been conducted in many parts of the world usually without using molecular tools nor the rigor included in this paper. The authors are commended for their attention to detail. Such a paper should hopefully elevate similar studies conducted in other parts of the world.*

*Reviewer #2: (No Response)*

*PLOS authors have the option to publish the peer review history of their article (*[*what does this mean?*](https://track.editorialmanager.com/CL0/https:%2F%2Fjournals.plos.org%2Fplosntds%2Fs%2Feditorial-and-peer-review-process%23loc-peer-review-history/1/010f0193b97b5b2f-95136d03-92aa-4f16-a13c-af099196f883-000000/o3YDcnQ7Ow6Ij-Io3jYx24GsfVSleS3h7E-2Zx1HZO0=189)*). If published, this will include your full peer review and any attached files.

If you choose “no”, your identity will remain anonymous but your review may still be made public.*

***Do you want your identity to be public for this peer review?*** *For information about this choice, including consent withdrawal, please see our* [*Privacy Policy*](https://track.editorialmanager.com/CL0/https:%2F%2Fwww.plos.org%2Fprivacy-policy/1/010f0193b97b5b2f-95136d03-92aa-4f16-a13c-af099196f883-000000/aEm0FhUJOMu2lODlZwJiN1664cAZvMfpkD4l656i8Tw=189)*.*

*Reviewer #1: No*

*Reviewer #2:* ***Yes:*** *Donna Lynn Tyungu*

*[NOTE: If reviewer comments were submitted as an attachment file, they will be attached to this email and accessible via the submission site. Please log into your account, locate the manuscript record, and check for the action link "View Attachments". If this link does not appear, there are no attachment files.]*

**Figure resubmission:**

 While revising your submission, please upload your figure files to the Preflight Analysis and Conversion Engine (PACE) digital diagnostic tool, https://pacev2.apexcovantage.com/. PACE helps ensure that figures meet PLOS requirements. To use PACE, you must first register as a user. Registration is free. Then, login and navigate to the UPLOAD tab, where you will find detailed instructions on how to use the tool. If you encounter any issues or have any questions when using PACE, please email PLOS at figures@plos.org. Please note that Supporting Information files do not need this step. If there are other versions of figure files still present in your submission file inventory at resubmission, please replace them with the PACE-processed versions.

**Reproducibility:**

To enhance the reproducibility of your results, we recommend that authors of applicable studies deposit laboratory protocols in protocols.io, where a protocol can be assigned its own identifier (DOI) such that it can be cited independently in the future. Additionally, PLOS ONE offers an option to publish peer-reviewed clinical study protocols. Read more information on sharing protocols at https://plos.org/protocols?utm_medium=editorial-email&utm_source=authorletters&utm_campaign=protocols
